# The effect of combining prewarming with intraoperative phenylephrine infusion on the prevention of hypothermia in patients undergoing urological surgery: a prospective, randomized, and controlled trial

**DOI:** 10.7150/ijms.89671

**Published:** 2023-10-24

**Authors:** Sung-Ae Cho, Seok-Jin Lee, Jun-ho Kim, Woojin Kwon, Tae-Yun Sung

**Affiliations:** 1 Department of Anaesthesiology and Pain Medicine, Konyang University Hospital, Konyang University Myunggok Medical Research Institute, Konyang University College of Medicine, Daejeon, Korea.; 2 Department of Anaesthesiology and Pain Medicine, Konyang University Hospital, Konyang University College of Medicine, Daejeon, Korea.

**Keywords:** Temperature, Equipment, Hypothermia, Incidence, Phenylephrine, Urological Surgery.

## Abstract

**Background:** Hypothermia is common in patients undergoing urological surgery; however, no single preventative modality is completely effective. This study evaluated the effects of combining prewarming with intraoperative phenylephrine infusion for the prevention of hypothermia in patients undergoing urological surgery.

**Methods:** This prospective study enrolled 58 patients scheduled for urological surgery under general anesthesia. The patients were randomized into two groups (n = 29). Patients in the experimental (prewarming and phenylephrine infusion) group (PP group) received prewarming for 20 min and intraoperative phenylephrine infusion, whereas those in the control group (C group) received no active prewarming with only intermittent administration of vasoactive agents. The patient's sublingual temperatures before and after anesthesia and nasopharyngeal temperature during anesthesia were recorded as core temperatures.

**Results:** The incidence of intraoperative hypothermia was higher in the C group than in the PP group (57.7% [15/26] vs. 23.1% [6/26], *P* = 0.01). The severity of intraoperative hypothermia was higher in the C group than in the PP group (*P* = 0.004). The nasopharyngeal temperature at the end of surgery was lower in the C group than in the PP group (35.8 ± 0.6°C vs. 36.3 ± 0.4°C, *P* = 0.002). The trend of core temperature decline during the first hour after anesthesia induction differed between the two groups (*P* = 0.003; its decline was more gradual in the PP group).

**Conclusions:** The combination of prewarming for 20 min and intraoperative phenylephrine infusion reduced the incidence and severity of intraoperative hypothermia and modified the trend of decreasing core temperatures in patients undergoing urological surgery.

## Introduction

Perioperative hypothermia (core temperature < 36.0°C) is common in urological surgeries requiring large amounts of irrigation fluids and occurs in approximately 88% of patients 1,2. Even a 1-2°C drop in core temperature increases the risks of wound infection, blood loss, transfusion, morbid myocardial events, and prolonged hospitalization. Therefore, maintaining normothermia during the perioperative period is essential for improving surgical outcomes and patient safety 3-5.

Without interventions to prevent hypothermia, the core body temperature typically decreases by 0.5-1.5°C for the first hour after induction of general anesthesia, mainly due to the internal core-to-peripheral redistribution of body heat 3,4,6; this redistribution contributes to 81% of the reduction in core temperature during the first hour after anesthesia induction 7. The best way to prevent redistribution hypothermia is to apply active warming to the skin surface before anesthesia induction (i.e., prewarming) 8. While short-term prewarming for only 10-20 min is effective in preventing hypothermia in patients undergoing minor surgery under general anesthesia 9, prewarming for 20 min in patients undergoing urological surgery under spinal anesthesia is insufficient to prevent hypothermia 1, and its effects on the incidence of intraoperative hypothermia are conflicting 1,10. These previous findings suggest that patients undergoing urological surgery require additional strategies in addition to prewarming to prevent hypothermia. Other means that can reduce redistribution hypothermia include intraoperative infusion of phenylephrine, bolus phenylephrine prior to propofol, induction of anesthesia with etomidate, ketamine, and sevoflurane, fructose administration, amino acid infusions, and the administration of vasodilators before anesthesia 8,11-17. Among these, intraoperative infusion of phenylephrine, a pure α_1_-adrenoceptor agonist, reduces the magnitude of redistribution hypothermia in patients under general or spinal anesthesia 11,18.

In patients undergoing urological surgery, maintaining perioperative normothermia is difficult with only a single-modality intervention owing to the use of large volumes of irrigation fluids 1,10. Thus, a multimodal approach is required. However, few studies have evaluated the efficacy of combined thermal interventions in preventing hypothermia 1,12. Our hypothesis was that combining prewarming with intraoperative phenylephrine infusion contributes to maintaining normothermia in patients undergoing urological surgery under general anesthesia. Therefore, this study evaluated the effects of the combination of prewarming and intraoperative phenylephrine infusion. The primary outcome was the incidence of intraoperative hypothermia and the main secondary outcome was the severity of intraoperative hypothermia.

## Materials and Methods

This prospective randomized controlled study was approved by the Institutional Review Board (KYUH 2021-08-007) of our hospital and registered at the Korean Clinical Research Information Service (https://cris.nih.go.kr/, KCT0006599). This study was conducted between February 2021 and January 2023 at a single university hospital after obtaining written informed consent from the participants and/or their legal surrogates.

We included patients aged 19-79 years with an American Society of Anesthesiologists Physical Status I-II who underwent elective urological surgery with an expected surgical duration of ≥ 60 min. The exclusion criteria were surgery involving large amounts of bleeding or transfusions were expected; surgery requiring carbon dioxide insufflation in the abdominal cavity; a patient preoperative body temperature of < 36.0°C or > 37.5°C; a patient body mass index of > 35 kg/m^2^; severe endocrine, cardiovascular, or respiratory disease; psychiatric disease or cognitive impairment; and hemodynamic instability upon admission to the operating room (bradycardia [heart rate (HR) < 45 beats/min], tachycardia [HR > 120 beats/min], uncontrolled hypertension [systolic blood pressure (SBP) > 180 mmHg], or hypotension [SBP < 90 mmHg]). Additionally, to evaluate the effect of the combination of prewarming and intraoperative phenylephrine infusion on temperature decline trends during the first hour after induction of anesthesia, patients whose operation time was less than 1 h were excluded from this study.

The patients were randomly assigned to one of two groups at a 1:1 ratio by an assistant unrelated to this study using online randomization software (Researcher Randomizer; www.randomizer.org) and the patient's group allocation was concealed in sealed opaque envelopes. The patients in the experimental (prewarming and phenylephrine infusion) group (PP group) received active prewarming for 20 min using a forced-air warming device and intravenous phenylephrine infusion during anesthesia, while those in the control group (C group) received usual care (i.e., no active prewarming and intermittent administration of vasoactive agents depending on blood pressure and HR). Upon patient arrival at the preoperative holding area, an anesthesia resident not involved in data collection opened an opaque, sealed envelope containing the patient's group allocation.

At our hospital, the ambient temperatures of the preoperative holding area and post-anesthesia care unit (PACU) were adjusted to a target range of 22-25°C. The ambient operating room temperature was measured using a digital thermometer (Model No. TH01D, Kyungin, South Korea) mounted on a wall away from a cooling or heating unit and recorded at the start and end of the surgery.

The patients fasted for at least 8 hrs and did not receive premedication. In the preoperative holding area, their sublingual temperature was measured by a trained anesthesiology resident by placing the probe (Welch-Allyn SureTemp Plus Electronic Thermometer Model 692, Welch Allyn Inc., Skaneateles Falls, NY, USA; accurate to ± 0.1°C for patient temperatures in the range of 26.7-43.4°C) under the tongue in the posterior sublingual pocket lateral to the center of the lower jaw. Thereafter, patients in the C group received standard preoperative passive insulation using a cotton blanket, whereas those in the PP group received active warming using a forced-air blanket (Bair Hugger^TM^ Full Body Blanket Model 30000; Arizant Healthcare Inc., Eden Prairie, MN, USA) placed over the entire body and covered with a cotton blanket. The set temperature of the forced-air warmer was 43°C (“high”), although the temperature was adjusted to 38°C (“medium”) if patients complained that it was too warm. After 20 min of warming, the forced-air warming device was turned off and the patients were transferred to the operating room with a cotton blanket over a forced-air blanket.

Upon arrival in the operating room, standard monitoring, including pulse oximetry, electrocardiography, non-invasive automated blood pressure measurement, patient state index (PSI; SedLine®; Masimo Corp., Irvine, CA, USA) assessment, and acceleromyography (TOF-Watch SX^®^; Organon Ltd., Dublin, Ireland), were performed. Before anesthesia induction, baseline blood pressure and HR were measured. General anesthesia was induced using propofol and fentanyl, and endotracheal intubation was facilitated using rocuronium. Immediately after endotracheal intubation, a nasopharyngeal thermistor probe (L000412, Gonimed Co., South Korea) was inserted at a depth of 10-20 cm from the nares to measure the core temperature 4. Subsequently, in the operating room and PACU, the body temperature, blood pressure, and HR were recorded every 15 min.

Anesthesia was maintained with desflurane and 50% nitrous oxide (0.8-1.2 of age-adjusted minimum alveolar concentration) to maintain the PSI at 25-50. In the PP group, phenylephrine infusion was initiated at an initial dose of 0.3 μg/kg/min immediately after anesthesia induction and allowed up to 0.7 μg/kg/min as long as the SBP did not exceed 120% of the preanesthetic SBP (baseline). The phenylephrine infusion was maintained until the end of surgery at a dose range of 0.3-0.7 μg/kg/min. In contrast, in the C group, phenylephrine was administered intermittently at 50-100 μg when the SBP decreased to < 80% of the baseline. In both groups, nicardipine (0.5 mg) was administered intravenously if the SBP exceeded 120% of the baseline or 180 mmHg. When the SBP decreased to < 80% of the baseline and the HR was < 50 beats/min, ephedrine (5-10 mg) was administered intravenously. When the HR was < 45 beats/min or > 120 beats/min, atropine (0.5 mg) or esmolol (10 mg) was administered.

At the end of surgical preparation, including positioning, surgical scrubbing, and draping, all patients received forced-air warming on their upper body at a set temperature of 38°C throughout the surgery. During anesthesia, inhaled gas was supplied through a respiratory circuit heated to 39.5°C and humidified, and intravenous and irrigation fluids were administered at room temperature. After anesthesia, the volumes of intravenous and irrigation fluids and blood loss estimated by the surgeon were recorded.

In the PACU, forced-air warming (set temperature 43°C) was applied when the patient's body temperature was < 36°C or the patient complained of feeling cold or when shivering was observed. If shivering was present, 25 mg meperidine was administered intravenously. Postoperative pain was evaluated using a numeric rating scale (NRS; 0 = no pain, 10 = worst pain imaginable); if an NRS score of > 4 and analgesics were requested, 0.5-1 μg/kg of fentanyl was administered intravenously. All adverse events occurring in the PACU were recorded.

In this study, hypothermia was defined as a sublingual or nasopharyngeal temperature of < 36°C, and the hypothermia severity was graded as mild (35.5-35.9°C), moderate (35.0-35.4°C), or severe (34.5-34.9°C) 10. If the nasopharyngeal temperature measured during anesthesia decreased below 36°C even once, it was considered intraoperative hypothermia. The incidence of intraoperative hypothermia (nasopharyngeal temperature < 36°C) was compared as the primary outcome. The secondary outcomes were the severity of intraoperative hypothermia, nasopharyngeal temperature at the end of surgery, incidence of shivering, number of patients receiving active warming in the care unit (PACU), and changes in core temperature during the first hour after anesthesia induction. All outcome variables were collected by an anesthesiology resident who was not involved in patient care and was blinded to the purpose of this study.

### Statistical analyses

In a preliminary study (n = 12 per group), the incidence rates of intraoperative hypothermia in the PP and C groups were 25% (3/12) and 66.7% (8/12), respectively. The sample size was calculated with an effect size *h* of 0.840, an α value of 0.05 (two-tailed), a power of 0.8, and an allocation ratio of 1:1; therefore, 26 patients were required per group. We enrolled 29 patients in each group to compensate for a potential dropout rate of 10%. The sample size was calculated using G*Power software (version 3.1.9.7; Franz Faul, Universitat Kiel, Germany).

Continuous variables were analyzed using Student's t- or Mann-Whitney U test after assessing the data distribution using the Kolmogorov-Smirnov test and are presented as the mean ± standard deviation or median (interquartile range). Categorical variables were compared using the χ^2^ test, χ^2^ test for trends (linear-by-linear association), or Fisher's exact test, as appropriate and expressed as numbers (%) or numbers. Repeatedly measured variables (e.g., temperature, SBP, and HR) were analyzed using repeated-measures analysis of variance with Bonferroni correction. In all analyses, a two-sided *P*-value < 0.05 was considered statistically significant. Cohen's effect sizes *d* and *h* were used to compare continuous and categorical variables, respectively. Statistical analyses were performed using IBM SPSS Statistics for Windows, version 27.0 (IBM Corp., Armonk, NY, USA).

## Results

**Figure 1:**
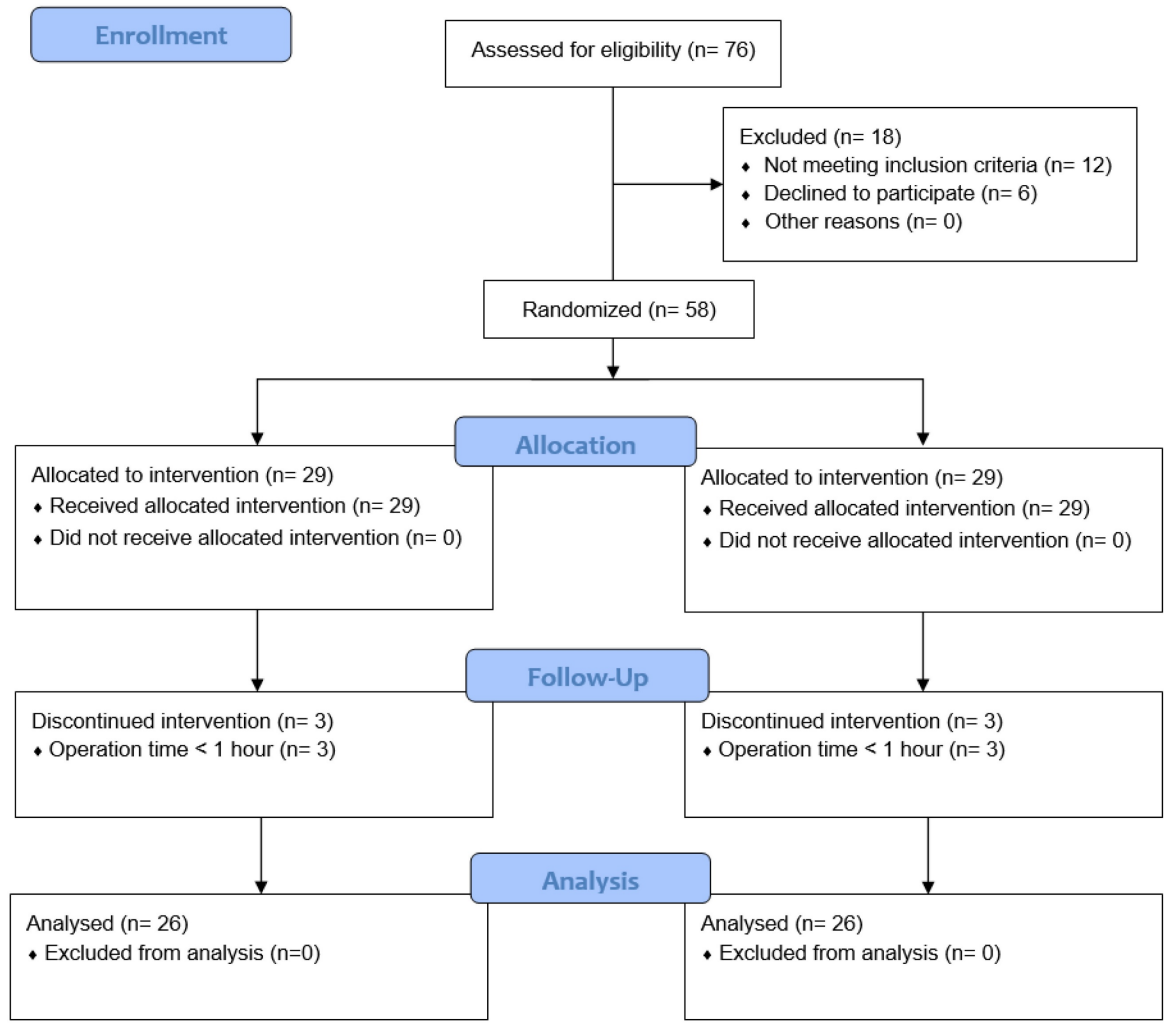
** Study flow chart.** C group: control group; PP group: prewarming and phenylephrine infusion group.

Of 76 screened patients, 18 were excluded; thus, the remaining 58 patients were assigned to the two study groups. Three patients in each group were withdrawn from the study because their operation time was < 1 h. Thus, 26 patients in each group completed the study (Fig. 1). There were no statistically significant differences in patient characteristics or baseline data between the groups (Table 1). Types of surgery also did not differ between the groups (Table 2).

Table 3 presents details of the prewarming and vasoactive drugs administered during anesthesia. In the PP group, 1 (3.8%) patient complained of being too warm during prewarming and the set temperature was adjusted from 43°C to 38°C. The times (mean ± standard deviation) from the end of prewarming to the induction of anesthesia and resumption of intraoperative forced-air warming were 9.1 ± 3.6 and 22.5 ± 6.5 min, respectively. The number of patients administered phenylephrine did not differ between the groups, although the dose (median [interquartile range]) of phenylephrine was significantly higher in the PP group than in the C group (3115.5 [2376 - 4409.8] μg vs. 200 [50 - 612.5] μg, *P* < 0.001). The levels of ephedrine, nicardipine, esmolol, and atropine did not differ between the groups.

Table 4 presents the outcome data during the study period. As the primary outcome, the incidence of intraoperative hypothermia was higher in the C group than in the PP group (57.7% [15/26] vs. 23.1% [6/26]; relative risk [RR] 2.26; 95% confidence interval [CI] for RR 1.09 to 4.66; effect size *h* = 0.72; *P* = 0.01). The severity of intraoperative hypothermia was higher in the C group than in the PP group (*P* = 0.004). The nasopharyngeal core temperature at the end of surgery was lower in the C group than in the PP group (35.8 ± 0.6°C vs. 36.3 ± 0.4°C; mean difference [MD] -0.4; 95% CI for MD -0.7 to -0.2; effect size *d* = 0.98; *P* = 0.002). In the PACU, the number of patients requiring active warming was higher in the C group than in the PP group (57.7% [15/26] vs. 26.9% [7/26]; MD 30.8%; 95% CI for MD 4.0% to 52.1%; effect size *h* = 0.64; *P* = 0.03). However, the incidence of shivering, NRS score for pain, and requirement for analgesics were similar between the groups.

**Figure 2:**
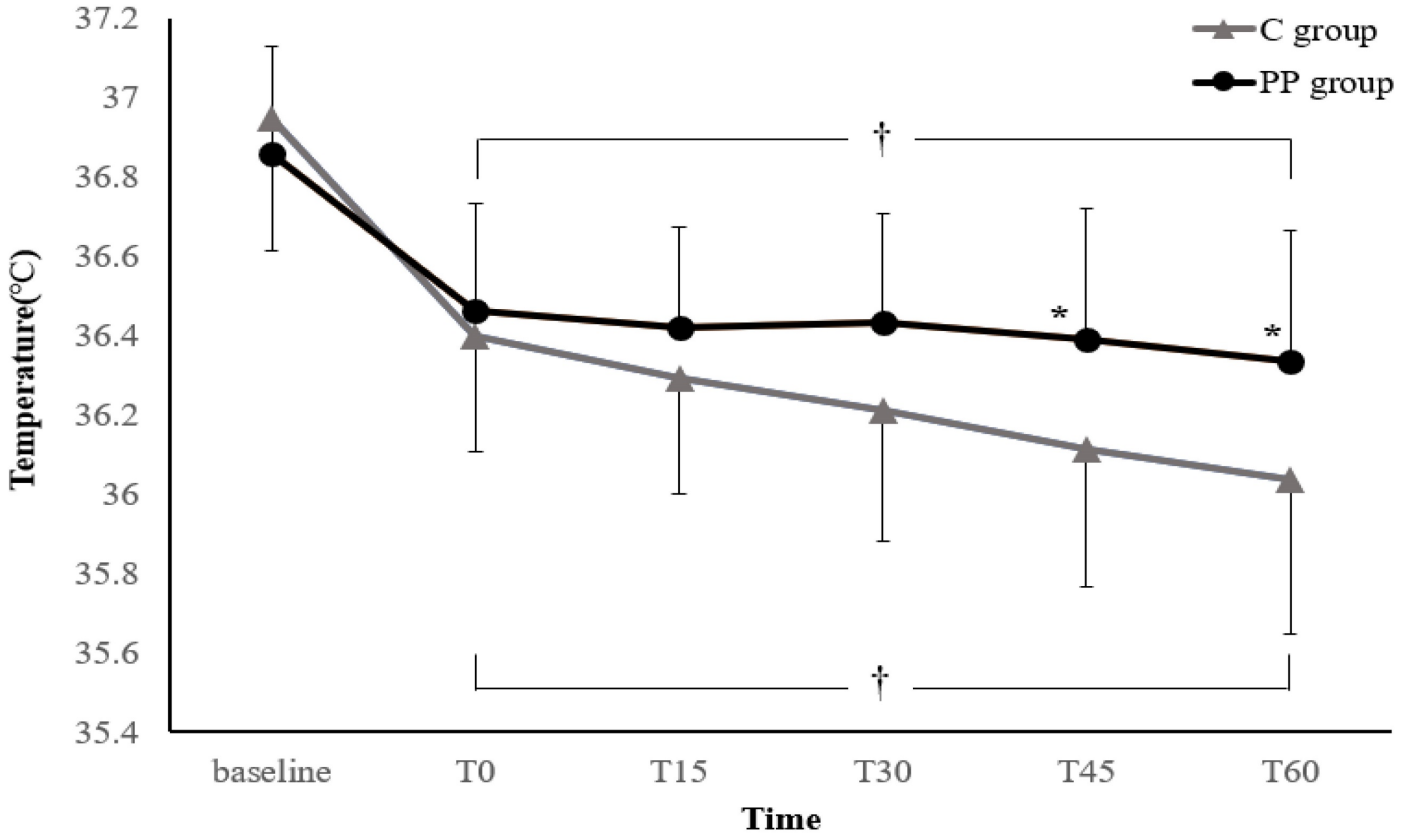
** Changes in core temperature during the first hour after anesthesia induction.** Values are expressed as the mean ± standard deviation. Baseline: arrival in the preoperative holding area; T0-60: immediately to 60 min after anesthesia induction; C group: control group; PP group: prewarming and phenylephrine infusion group. ^*^P < 0.05, vs. C group (Bonferroni-corrected). ^†^*P* < 0.05, vs. baseline in each group (Bonferroni-corrected).

Figure 2 illustrates the changes in core temperature during the first hour after anesthesia induction. In both groups, the core temperature decreased after anesthesia induction to below the baseline temperature. However, the treatment-by-time interaction was significant, suggesting that the trend in core temperature change differed significantly between the two groups (*P* = 0.003). In addition, the core temperatures at 45 and 60 min after anesthesia induction were significantly higher in the PP group than in the C group (Bonferroni-corrected *P* < 0.05).

**Figure 3:**
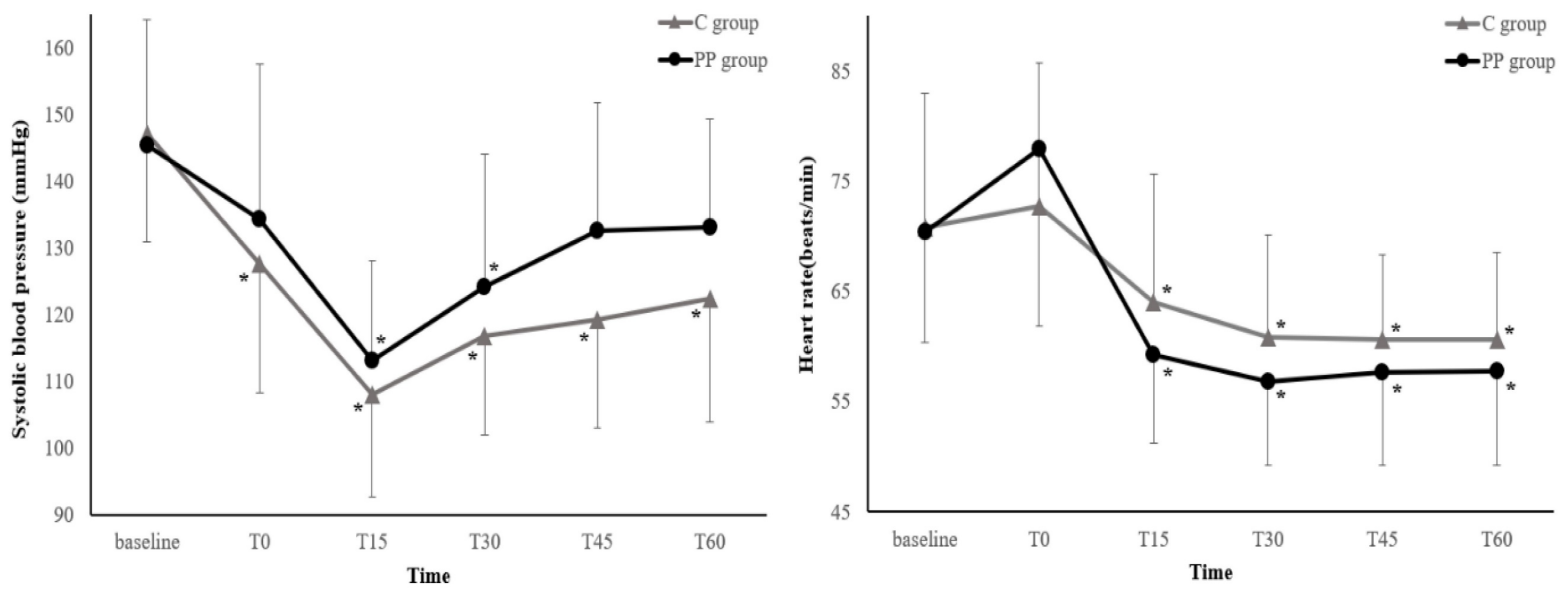
** Changes in the systolic blood pressure and heart rate during the first hour after induction of anesthesia.** Values are expressed as the mean ± standard deviation. Baseline: before anesthesia induction; T0-60: immediately to 60 min after induction of anesthesia; C group: control group; PP group: prewarming and phenylephrine infusion group. Neither the systolic blood pressure nor heart rate differed between groups at any time point (all *P* > 0.05, Bonferroni-corrected). ^*^P < 0.05, vs. baseline in each group (Bonferroni-corrected).

Figure 3 illustrates the changes in the SBP and HR during the first hour after anesthesia induction. The SBP and HR trends did not differ between the two groups (*P* = 0.09 and 0.40, respectively). Neither the SBP nor HR showed any differences between the groups at any measurement points (all *P* > 0.05, Bonferroni-corrected).

Adverse events also did not differ between the groups (Table 5).

## Discussion

This study evaluated the effect of combined prewarming and intraoperative phenylephrine infusion on reducing hypothermia in patients undergoing urological surgery under general anesthesia. The primary outcome (incidence of intraoperative hypothermia) and predominant secondary outcomes (severity of intraoperative hypothermia, changes in temperature during the first hour after anesthesia induction, nasopharyngeal temperature at the end of surgery, and number of patients receiving active warming in the PACU) differed significantly between the two groups. Our findings suggest that this multimodal intervention strategy may be effective in preventing or reducing hypothermia in patients undergoing urological surgery.

While various methods have been proposed to prevent hypothermia, no single-modality intervention can completely prevent hypothermia, and no clear protocol exists 19. Cold irrigation fluids are among the important causes of heat loss in patients undergoing urological surgery 1,10, and clinical practice guideline recommends the use of irrigation fluids prewarmed to 38-40°C 20. However, the preventive effect of warmed irrigation fluids on intraoperative hypothermia is controversial 19. Additionally, the risk of tissue burning due to overwarming of the irrigation fluid should be considered 1. The use of warmed intravenous fluids using an infusion warming device may also help prevent hypothermia but is not effective at low flow rates (< 500 ml/h) 20. Although active forced-air warming is safer and more effective in preventing hypothermia than other warming methods (e.g., passive insulation and circulating-water mattresses) 8,21, intraoperative forced-air warming alone does not compensate for the initial hypothermia by redistribution following the anesthesia induction, and hypothermia reportedly occurred in 64% of patients 45 min after the induction of general anesthesia 5. Similarly, intraoperative hypothermia occurred in 57.7% of the patients in the C group in this study. In contrast, in the PP group, intraoperative phenylephrine infusion following prewarming modified the trend of the initial decline in core temperature during the first hour after anesthesia and reduced the hypothermia incidence and severity.

Prewarming and intraoperative warming are recommended because intraoperative warming alone is often ineffective in maintaining perioperative normothermia 20,21. Prewarming has little effect on core temperature but increases peripheral temperature and total body heat content. Consequently, the core-to-peripheral temperature gradient is reduced, which contributes to the prevention of redistribution hypothermia by reducing the core-to-peripheral heat flow after anesthesia 3,4,21. However, the efficacy of prewarming decreases with increasing time between prewarming and resumption of intraoperative warming. For each minute delay, the risk of intraoperative hypothermia increases by 4.9% 22, and interruptions of warming of > 20 min significantly increases the incidence of hypothermia compared with interruptions of < 20 min 23. In this study, warming was stopped during transfer to the operating room after prewarming, induction of anesthesia, and surgical preparation, with an average duration of warming interruption of 22.5 min. This is similar to the time of warming interruption in previous studies 23-25. In a study of patients undergoing ureteroscopic stone surgery, the average time interval from prewarming stop to intraoperative rewarming was 20.5 min in adult patients (aged 20-50 years) and 22.1 min in older patients (> 65 years) 24. In another study of patients undergoing laparoscopic or open urological surgery, an average of 22 min was required between anesthesia induction and surgery start, which prevented effective thermal intervention 25. Especially after anesthesia induction, it would be difficult to apply a forced-air warming blanket during surgical positioning and scrubbing. However, because most of this warming interruption period corresponds to the time when anesthetic-induced hypotension and a rapid decrease in core temperature occur simultaneously, thermoprotective interventions other than forced-air warming are required; this, the present study implemented phenylephrine infusion.

Phenylephrine, a pure synthetic vasoconstrictor, increases blood pressure via arteriolar vasoconstriction, which in turn results in a baroreceptor-triggered reduction in the HR and cardiac output 26. The preventive effects of phenylephrine on hypothermia can be explained by its pharmacological properties. Phenylephrine may attenuate the magnitude of redistribution of hypothermia by maintaining vasoconstriction of the precapillary vasculature 11. Additionally, a decrease in the cardiac output may limit the convective transfer of heat to peripheral tissues 27. Vasoconstriction by phenylephrine reduces cutaneous perfusion, which may result in decreased flow of metabolic heat to the environment 28.

Previous studies on patients under spinal anesthesia reported that either prewarming for 20 min 1 or intraoperative phenylephrine infusion 29 reduced the incidence of intraoperative hypothermia compared with usual care. However, these studies used only one of the two methods, and more than half of the patients developed hypothermia. In contrast, in this study, which applied both methods, hypothermia occurred in less than one-quarter of the patients. In addition, compared with the finding of the previous two studies, the significant reduction in the incidence of hypothermia in this study is more meaningful because it occurred in patients under general anesthesia, with more severe impairment of central thermoregulation 6.

In this study, hemodynamic parameters (e.g., SBP and HR) were adjusted within the same hemodynamic target range during surgery in all patients. Although the dose of phenylephrine was higher in the PP group due to continuous infusion of phenylephrine, the number of patients receiving phenylephrine was comparable in both groups, and other vasoactive drugs showed no differences between the two groups in both dose and number of patients administered. Consequently, the SBP and HR did not show statistical significance between the groups at all measurement points. This may have partly contributed to the prevention of redistribution hypothermia in the C group. The incidence of hypothermia in the C group in our study (57.7%) was comparable to that in the group in which either prewarming (56%) or phenylephrine infusion (57.3%) was applied in previous studies 1,29. In contrast, in the C group, the SBP decreased at all measurement points compared with baseline, whereas in the PP group, the SBP decreased only at 15 and 30 min after anesthesia, indicating that continuous rather than intermittent bolus administration phenylephrine infusion was more effective in preventing both redistribution hypothermia and hypotension by inhibiting vasodilation. Meanwhile, although there was no statistical difference in SBP at all measurement points, the trend of SBP was higher in the PP group, thus caution may be necessary depending on the target patient.

This study has limitations. Because intraoperative forced-air warming is a routine intervention applied to all patients undergoing surgery under general anesthesia according to our institutional protocol, it was applied to all patients in both groups in this study. This may have affected the outcomes of this study. However, intraoperative forced-air warming reduces convective and radiant heat losses and may contribute to an increase in body heat content 3,8, but it did not compensate for the reduction in core temperature caused by redistribution during the first hour after induction of anesthesia 5. Therefore, considering the mean operation times in this study (65 min in the C group and 77.5 min in the PP group), the impact on outcomes, such as the incidence and severity of intraoperative hypothermia and changes in temperature of hypothermia during the first hour after induction of anesthesia, would not have been significant. Second, although there was no statistically discernible difference in the amount of irrigation fluid between the two groups, the wide variability in the amount of irrigation fluid could be a weakness of this study. Because the amount of irrigation fluid is one of the important causes of intraoperative hypothermia 4, there is concern about the possibility that the amount of irrigation fluid may have influenced the results of this study.

In conclusion, a combination of prewarming for 20 min and intraoperative phenylephrine infusion reduced the incidence and severity of intraoperative hypothermia and modified the trend of core temperature reduction in patients undergoing urological surgery.

## Figures and Tables

**Table 1:** Patient characteristics and perioperative data.

Variable	C group (n = 26)	PP group (n = 26)	*P*
Age (years)	56.0 ± 13.3	59.9 ± 12.3	0.28
Sex (male/female)	17/9	20/6	0.54
Weight (kg)	70.7 ± 15.5	69.0 ± 9.5	0.64
Height (cm)	164.9 ± 7.7	165.4 ± 7.9	0.81
Body mass index (kg/m^2^)	25.8 ± 4.2	25.2 ± 2.6	0.52
ASA physical status (I/II)	1/25	2/24	> 0.99
Surgical position			> 0.99
Lithotomy	22 (84.6%)	21 (80.8%)	
Supine	4 (15.4 %)	5 (19.2%)	
Baseline temperature (°C)	36.9 (36.7-37.2)	36.9 (36.7-37.0)	0.50
Baseline SBP (mmHg)	147.2 ± 16.3	145.5 ± 18.8	0.72
Baseline HR (beat/min)	70.8 ± 12.1	70.3 ± 10.0	0.87
Operating room temperature			
Start of surgery (°C)	22.8 ± 1.0	22.4 ± 1.2	0.18
End of surgery (°C)	23.5 ± 1.0	23.1 ± 1.0	0.16
Intravenous fluid (ml)	400 (287.5-500.0)	350.0 (287.5-412.5)	0.41
Irrigation fluid (ml)	3000 (1575-9000)	1650 (362.5-9750)	0.45
Estimated blood loss (ml)	5.0 (1.0-10.0)	7.5 (2.0-30.0)	0.39
Duration of surgery (min)	65.0 (53.8-85.0)	77.5 (53.8-124.0)	0.41
Duration of anesthesia (min)	92.5 (80.0-112.5)	101.0 (80.8-114.8)	0.39

Data are expressed as the mean ± standard deviation, median (interquartile range), number, or number (%). C group: control group; PP group: prewarming and phenylephrine infusion group; ASA: American Society of Anesthesiologists; SBP: systolic blood pressure; HR: heart rate.

**Table 2:** Urological procedures.

Variable	C group (n = 26)	PP group (n = 26)	P
Type of surgery			0.56
Ureteroscpic litholapaxy	12 (46.2%)	10 (38.5%)	0.58
TURP	7 (26.9%)	7 (26.9%)	1.00
TURBT	3 (11.5%	4 (15.4%)	> 0.99
Hydrocelectomy	2 (7.7%)	3 (11.5%)	> 0.99
Radical orchiectomy	1 (3.8%)	1 (3.8%)	1.00
Percutaneous nephrolithotomy	1 (3.8%)	0	> 0.99
Vasovasostomy	0	1 (3.8%)	> 0.99

Data are expressed as numbers (%). C group: control group; PP group: prewarming and phenylephrine infusion group; TURP: transurethral resection of the prostate; TURBT: transurethral resection of bladder tumors.

**Table 3:** Details of prewarming and vasoactive drugs administered during anesthesia.

Variable	C group (n = 26)	PP group (n = 26)	*P*
**Prewarming**			
Changed set prewarming temperature from 43°C to 38°C	NA	1 (3.8%)	NA
Time from the stop of prewarming to anesthesia induction (min)	NA	9.1 ± 3.6 7.5 (6-12.3)	NA
Time from the stop of prewarming to the start of intraoperative warming (min)	NA	22.5 ± 6.5 21 (19-24)	NA
**Vasoactive drugs**			
Phenylephrine			
Amount (μg)	200 (50-612.5)	3115.5 (2376-4409.8)	< 0.001
n (%)	22 (84.6%)	26 (100%)	0.11
Ephedrine			
Amount (mg)	0 (0-6.3)	0 (0-1.3)	0.12
n (%)	11 (42.3%)	6 (23.1%)	0.14
Nicardipine			
Amount (mg)	0 (0-0)	0 (0-0.1)	0.05
n (%)	1 (3.8%)	6 (23.1%)	0.10
Esmolol			
Amount (mg)	0 (0-0)	0 (0-0)	0.08
n (%)	0	3 (11.5%)	0.24
Atropine			
Amount (mg)	0 (0-0)	0 (0-0)	0.56
n (%)	2 (7.7%)	1 (3.8%)	> 0.99

Data are expressed as the mean ± standard deviation, median (interquartile range), or number (%). C group: control group; PP group: prewarming and phenylephrine infusion group; NA: not applicable.

**Table 4:** Outcome data during the study period.

Variable	C group (n = 26)	PP group (n = 26)	RR or MD (95% CI)	Effect size *d* or *h*	P
**In the operating room**					
Incidence of hypothermia	15 (57.7%)	6 (23.1%)	2.26 (1.09, 4.66)	0.72	0.01
Severity of hypothermia					0.004
Mild (35.5-35.9°C)	10 (38.5%)	6 (23.1%)	NA	0.34	
Moderate (35.0-35.4°C)	4 (15.4%)	0	NA	0.81	
Severe (34.5-34.9°C)	1 (3.8%)	0	NA	0.39	
Core temperature at the end of surgery (°C)	35.8 ± 0.6	36.3 ± 0.4	-0.4 (-0.7, -0.2)	0.98	0.002
**In the PACU**					
Incidence of shivering	0	1 (3.8%)	NA	0.39	> 0.99
Active warming required	15 (57.7%)	7 (26.9%)	30.8% (4.0%, 52.1%)	0.64	0.03
NRS for pain (0-10)	2 (0-4.3)	0 (0-3.0)	NA	NA	0.30
Fentanyl	4 (15.4%)	4 (15.4%)	NA	0	1.00

Data are expressed as the number (%), mean ± standard deviation, or median (interquartile range). C: control; PP: prewarming and phenylephrine infusion; RR: relative risk; MD: mean difference; PACU: post-anesthesia care unit; NRS: numeric rating scale (0 = no pain, 10 = worst pain imaginable).

**Table 5:** Adverse events.

Variable	C group (n = 26)	PP group (n = 26)	*P*
CRBD	4 (15.4%)	2 (7.7%)	0.67
Sore throat	2 (7.7%)	2 (7.7%)	1.00
Nausea	1 (3.8%)	2 (7.7%)	> 0.99
Dizziness	1 (3.8%)	1 (3.8%)	1.00
Sputum	0	1 (3.8%)	> 0.99
Headache	0	1 (3.8%)	> 0.99
Hypotension	0	1 (3.8%)	> 0.99

Data are expressed as numbers (%). C group: control group; PP group: prewarming and phenylephrine infusion group; CRBD: catheter-related bladder discomfort.
